# Manipulating of Different-Polarized Reflected Waves with Graphene-based Plasmonic Metasurfaces in Terahertz Regime

**DOI:** 10.1038/s41598-017-10726-y

**Published:** 2017-09-05

**Authors:** Li Deng, Yongle Wu, Chen Zhang, Weijun Hong, Biao Peng, Jianfeng Zhu, Shufang Li

**Affiliations:** 1 Beijing Key Laboratory of Network System Architecture and Convergence, School of Information and Communication Engineering, Beijing University of Posts and Telecommunications, P.O. Box. 282, 100876 Beijing, China; 2 Beijing Key Laboratory of Work Safety Intelligent Monitoring, School of Electronic Engineering, Beijing University of Posts and Telecommunications, P.O. Box. 282, 100876 Beijing, China

## Abstract

A graphene-based plasmonic metasurface which can independently control different polarized electromagnetic waves with reasonably small losses in terahertz regime is proposed and demonstrated in this paper. This metasurface is composed of graphene based elements. Owing to anisotropic plasmonic resonance of the graphene-based elements, the reflected phases and magnitudes of orthogonally polarized waves can be independently controlled by varying dimensions of the element. Four types of graphene-based plasmonic metasurfaces with different reflected phases distributions are synthesized and simulated, exhibiting diverse functions such as polarized beam splitting, beam deflection, and linear-to-circular polarization conversion. The simulation results demonstrate excellent performances as theoretical expectation. The proposed graphene-based plasmonic metasurface can be applied to realize extremely light-weight, ultra-compact, and high-performances electromagnetic structures for diverse terahertz applications.

## Introduction

Controlling polarized waves at will has become a desired research topic from microwave to optical applications. It can be widely utilized in systems such as polarization multiplexing^1^, imaging processing^2^, and high data rate communications^3^, etc. On the other hand, based on the general Snell’s law^4^, the metasurface has been created as an interface to produce discontinuous phase shifts, yielding anomalous refractions and reflections. It can pattern series of planar sub-wavelength structures to realize desired amplitude, phase, or polarization properties, offering extra, but important degrees of freedom to manipulate the electromagnetic waves. Many novel and exciting applications of metasurfaces have been proposed, such as wave-front shaping^5^, polarization-controlled plasmonic coupler^6^, reflect-array^7^, transmit-array^8^, photonic spin Hall effect^9^, holography^10, 11^, and analog computing^12^. Meanwhile, focusing on polarization controlling, prominent works such as polarization beam splitters^13^, cross-polarization converters^14^, and linear to circular converters^15^, have been presented. All above-mentioned researches exhibit that the metasurfaces can provide powerful controlling capability, low-cost manufacturing and extremely suitable for device integration. However, most metasurfaces are based on metallic sub-wavelength structures which become quite lossy in terahertz band^16^, limiting their applications. Therefore, it is still a huge problem to independently manipulate different polarized electromagnetic waves with reasonably small losses in the terahertz band.

Fortunately, graphene, a monolayer of carbon atoms arranged in a honeycomb lattice^17, 18^, has emerged as a promising, alternative candidate for terahertz applications^19–27^. Thanks to the two- dimensional (2-D) nature of graphene, graphene surface plasmons present extremely small wavelengths and tight field confinement, while maintaining quite small loss in the terahertz regime. Moreover, through chemical doping or electrical gating^28–34^, the mechanical, electronic, optical, and thermal properties of graphene are highly tunable, which is impossible or inefficient if metals are applied. Naturally, graphene is extended into metasurface, and several meaningful works on graphene-based metasurfaces are conceptually reported and demonstrated. Ref. 35 present efficient designs of graphene-based thin absorbers, which are capable of near-unity absorption of the incident electromagnetic waves in the terahertz regime. In ref. 36, it shows that giant cross-polarization conversion of terahertz wave is possible at the plasmon resonance in the graphene nanoribbon array without applying external DC magnetic field. Ref. 37 proposed graphene-based plasmonic metasurfaces to steer infrared light in specific ways. Ref. 38 demonstrates tunable dual-band asymmetric transmission for circularly polarized waves with a graphene planar chiral metasurface. However, these graphene-based metasurfaces are lack of consideration on independent controlling over differently polarized electromagnetic waves.

In this paper, we propose graphene-based plasmonic metasurfaces, which can manipulate the transverse electric (TE) and transverse magnetic (TM) reflected waves independently with reasonably small losses in the terahertz regime. The unit cells of this graphene-based plasmonic metasurface are series of rectangular graphene based patches, which has anisotropic responses for each of orthogonal polarizations (TE and TM waves). The normally incident waves are totally reflected by the metal-grounded plane on the bottom of metasurface, but the reflection phases of both TE and TM waves are controlled independently by changing the dimensions of anisotropic unit cells of metasurface. Based on the proposed metasurfaces, four kinds of functional devices are designed for polarization beam splitting, beam deflection, and linear-to-circular polarization conversion with a deflection angle.

## Results

### Design and theory

**Figure 1:**
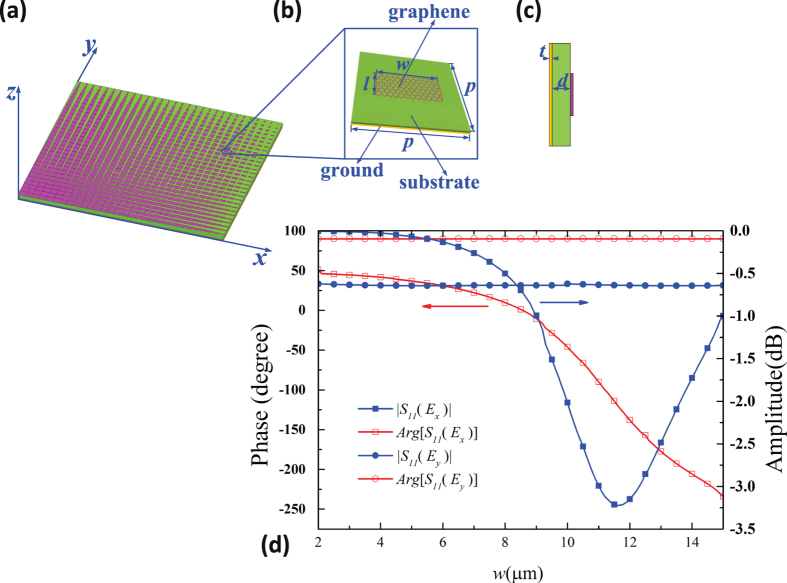
(**a**) The schematic of graphene based plasmonic metasurface, which can be generated by extending unit-cell along both of $x$ and $y$ directions. (**b**) The Fabry-Perot resonant unit element, which is composed of a rectangular graphene patch and a grounded quartz glasses (SiO_2_) substrate. (**c**) The side view of the unit element. (**d**) The reflected magnitudes and phases of the unit element, which shows that the TM wave reflected phases are gradually decreased from 50° to −240° with magnitudes larger than −3.3 dB when $w$ is increased from 2 *μ*m to 15 *μ*m. But the reflected magnitudes and phases will not be affected by changing $w$.

Graphene can strongly interact with electromagnetic waves in terahertz regime through plasmonic resonance^39, 40^. But for practical applications, wave-graphene interactions have to be further improved. Therefore, we design a Fabry-Perot resonant unit element, which is composed of a rectangular graphene patch and a square grounded quartz glasses (SiO_2_) substrate. When we periodically extent these elements along both of the $x$ and $y$ directions, as shown in Fig. 1(a), a 2-D graphene-based plasmonic metasurface can be generated. Incident terahertz wave can excite the plasmonic resonance of graphene patches on the top layer, and can be totally reflected by the bottom metallic ground. When a plane terahertz wave illuminates on the metasurface, the reflected fields from each interface interfere with each other. The top layer graphene patch array acts as a partially reflecting mirror, and the bottom metallic ground acts as a fully reflecting mirror, respectively. The dimensions of the element shown in Fig. 1(b) are $p$ = 15 *μ*m, and $w$ and $l$ can be changed independently to manipulate the reflection phases of TM and TE polarized electromagnetic waves, respectively. The reason is that the electric field in TM component of incident wave only can excite the plasmonic resonance in the $x$ direction, in contrast, for TE polarization, the electric field is parallel to the $y$ direction, and no plasmonic resonance can be excited in the $x$ direction. Thus, TM wave is only sensitive to the variation of $w$, in the $x$ direction, and similarly, TE wave is only sensitive to the variation of $l$, in the $y$ direction, respectively. The thickness of the quartz glasses (SiO_2_) spacer and bottom metallic ground plane are $d$ = 26 *μ*m and $t$ = 10 nm, respectively. The relative permittivity of the quartz glasses (SiO_2_) substrate is ${\varepsilon }_{r}\mathrm{=3.75}$, and the loss tangent is tan $\delta \mathrm{=0.0184}$ ^41^. Figure 1(c) demonstrates the side view of the proposed unit cell.

Figure 1(d) shows the reflection magnitudes and phases at 1.2 THz for TM and TE incident waves with fixed $l$ = 10 *μ*m and varied $w$ from 2 *μ*m to 15 *μ*m. It is demonstrated acceptable TM wave reflection magnitudes above −3.3 dB and reflection phases varied from 50° to −240°. Meanwhile, the TE wave reflection magnitudes and phases are kept in constant. If we change $l$ from 2 *μ*m to 15 *μ*m, and keep $w$ in constant, similarly, the TE wave reflection magnitudes are above −3.3 dB and reflection phases varied from 50° to −240°. Then the TM wave reflection properties will not be affected. Therefore, we conclude that the reflection properties of TM and TE polarized waves can be manipulated independently by varying lengths of $w$ and $l$, respectively. It is worth noting that, in practice, a phase range over 270° is sufficient to provide good performance^42^. Here, we cannot achieve a full phase range of 360°, the reason is that the intrinsic losses of graphene patch limits the phase shift range, and the structure performs as a damped oscillator. But we obtain a maximum phase range of 290° which is enough for practical applications. Furthermore, we can achieve reflectivity larger than −3.3 dB, enabling the design of novel functional graphene-based metasurfaces to steer reflected electromagnetic waves with acceptable efficiency. It is worth noting here that the plasmonic response of metals becomes less pronounced as operating in terahertz bands because of the weaker interaction between waves and electrons.Therefore, the metallic reflected metasurfaces operating in terahertz band often have reflectivity less than 30%^43–45^, which obviously have lower efficiency than our proposed graphene metasurface.

As above mentioned, we can utilize specific surface reflection phase distributions to manipulate electromagnetic waves arbitrarily. According to generalized laws of reflection and refraction^4^, we can introduce an in-plane phase gradient $d\phi /dx$ at the interface of two media. Thus, the relationship between the incident angle and reflection angle is^37^

1 $$sin({\theta }_{r})-\,\sin ({\theta }_{i})=\frac{\lambda }{2\pi \cdot {n}_{i}}\frac{d\phi }{dx}$$

where ${n}_{i}$ depicts the refractive index of the media at the incidence side, and ${\theta }_{i}$ and ${\theta }_{r}$ denote the incident and reflected angle, respectively. Specially, in the normal incidence situation and ${n}_{i}$ = 1, the reflection angle can be calculated as^37^

2 $${\theta }_{r}=si{n}^{-1}\frac{\lambda }{2\pi }\frac{d\phi }{dx}$$

Due to we can independently control TM and TE waves by changing only one dimensional parameters, we can easily extent the generalized laws of reflection into two dimensional cases, where the surface reflection phase distribution can be calculated as^15^

3 $${\phi }_{u}(x,y)={\phi }_{u}({x}_{0},{y}_{0})-\frac{2\pi }{{\lambda }_{0}}sin{\theta }_{u}[xcos({\varphi }_{u})+ysin({\varphi }_{u})],\quad u={E}_{x},{E}_{y}\mathrm{.}$$

where (${\varphi }_{{E}_{x}}$, ${\theta }_{{E}_{x}}$) and (${\varphi }_{{E}_{y}}$, ${\theta }_{{E}_{y}}$) depict the deflection directions of TM and TE waves, and ${\phi }_{u}({x}_{0},{y}_{0})$ depicts an arbitrary reference reflected phase. The desired ${\phi }_{u}(x,y)$ can be obtained by the graphene-based metasurface shown in Fig. 1. Simultaneously, the reflection phases of the TM and TE waves are dictated by changing the parameters $w$ and $l$, respectively. Therefore, we can independently manipulate the deflection angles of TM and TE waves according to Eq. (3).

### Simulation results

According to the proposed graphene-based element, We design four functional metasurfaces, named type 1, type 2, type 3, and type 4, respectively. Here, type 1 and type 2 act as two kinds of polarization beam splitters (PBSs), type 3 operates as a beam deflector, and type 4 is a linear-to-circular polarization converter, respectively. It is worth mention here that the ${\theta }_{{E}_{x}}$ or ${\theta }_{{E}_{y}}$ is defined as the angle between the TM or TE reflected polarized wave and the +$z$ axial, respectively. The range of $\theta$ is $(-{90}^{{\rm{o}}},{90}^{{\rm{o}}})$, negative angle means anticlockwise rotation from +$z$ axial. Similar, the ${\phi }_{{E}_{x}}$ or ${\phi }_{{E}_{y}}$ is defined as the angle between the projection of the TM or TE reflected wave in the XOY plane and the +$x$ axial, respectively, and the range of $\phi$ is ${\mathrm{(0}}^{{\rm{o}}},\,{360}^{{\rm{o}}})$. In all these four simulation models, we apply a normal incident plane wave as excitation, which has a frequency at 1.2 THz.

**Figure 2:**
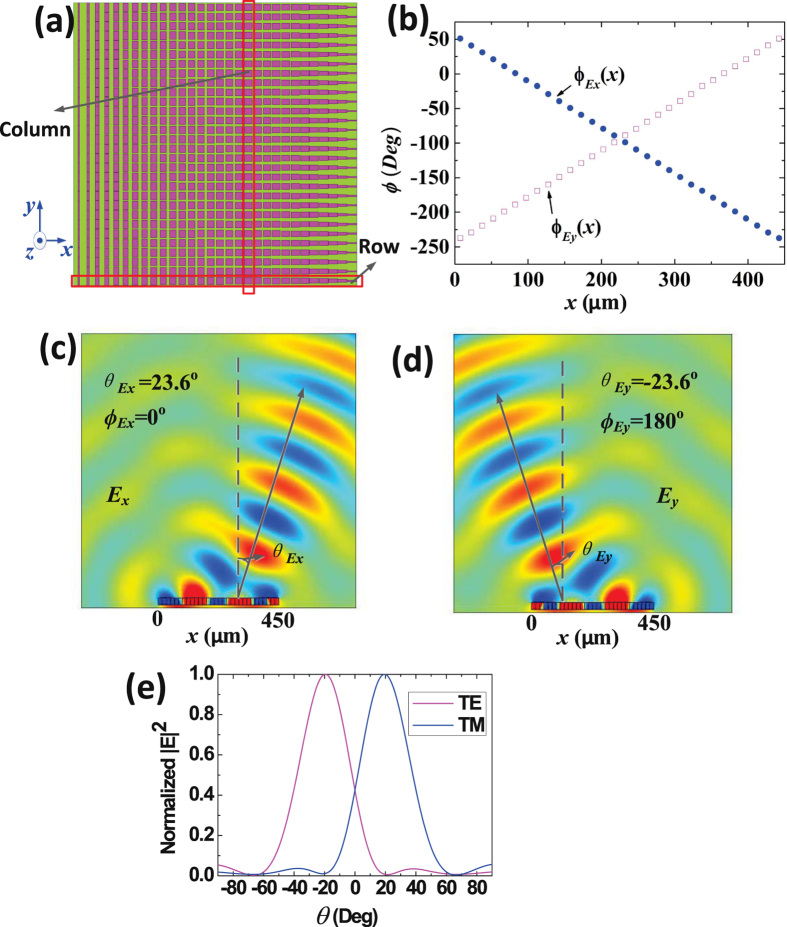
The structure and simulation results of type 1 graphene-based plasmonic metasurface. (**a**) The top view of the metasurface, which shows that the metasurface is made up of $30\times 30$ elements. The lengths of $w$ and $l$ of the elements in each row (along the $+x$ direction) are gradually increased and decreased, respectively, but the corresponding dimensions in each column (along the $+y$ direction) are unchanged. (**b**) The reflected phases distributions, which demonstrate that ${\phi }_{{E}_{y}}(x)$ and ${\phi }_{{E}_{x}}(x)$ are gradually decreased and increased along the $+x$ direction. (**c**) The electric-field distributions of TM reflected waves deflected to the direction of (${\varphi }_{{E}_{x}}\,=\,{0}^{{\rm{o}}}$, ${\theta }_{{E}_{x}}\,=\,{23.6}^{{\rm{o}}}$). (**d**) The electric-field distributions of TE reflected waves deflected to the direction of (${\varphi }_{{E}_{y}}\,=\,{180}^{{\rm{o}}}$, ${\theta }_{{E}_{y}}\,=\,-{23.6}^{{\rm{o}}}$). (**e**) Normalized far-field patterns of TM and TE reflected waves, in the XOZ plane.

**Figure 3:**
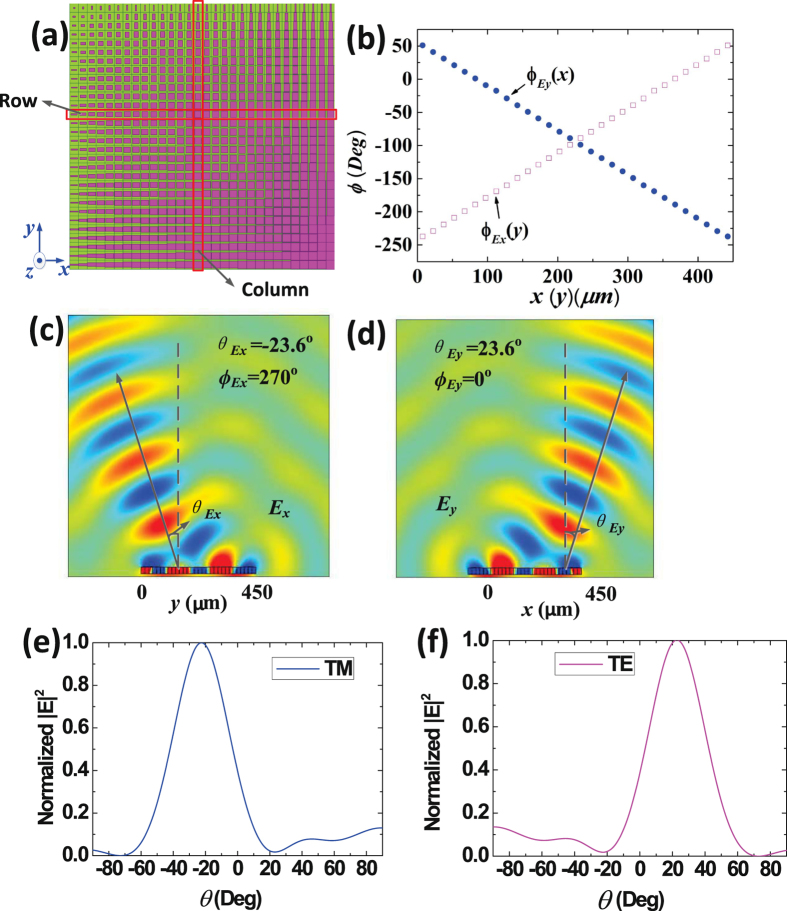
The structure and simulation results of type 2 graphene plasmonic metasurface. (**a**) The top view of the metasurface, which shows that the metasurface is made up of $30\times 30$ elements. The width $w$ is unchanged, and $l$ is gradually increased along the $+x$ direction; meanwhile, $w$ is gradually decreased and $l$ is unchanged along the $+y$ direction. (**b**) The reflected phases distributions, which demonstrate that ${\phi }_{{E}_{y}}(x)$ and ${\phi }_{{E}_{x}}(y)$ are gradually decreased and increased along the $+x$ direction and the $+y$ direction, respectively. (**c**) The electric-field distributions of TM reflected waves deflected to the direction of (${\varphi }_{{E}_{x}}\,=\,{270}^{{\rm{o}}}$, ${\theta }_{{E}_{x}}\,=-{23.6}^{{\rm{o}}}$). (**d**) The electric-field distributions of TE reflected waves deflected to the direction of (${\varphi }_{{E}_{y}}\,=\,{0}^{{\rm{o}}}$, ${\theta }_{{E}_{y}}\,=\,{23.6}^{{\rm{o}}}$). (**e**) Normalized far-field patterns of TM reflected waves, in the YOZ plane. (**f**) Normalized far-field patterns of TE reflected waves, in the XOZ plane.

Figures 2 and 3 show the designing and simulation results of type 1 and type 2 metasurfaces. As demonstrated in the Fig. 2, type 1 is designed to deflect the TM and TE waves to the directions of (${\varphi }_{{E}_{x}}\,=\,{0}^{{\rm{o}}}$, ${\theta }_{{E}_{x}}\,=\,{23.6}^{{\rm{o}}}$) and (${\varphi }_{{E}_{y}}\,=\,{180}^{{\rm{o}}}$, ${\theta }_{{E}_{y}}\,=\,-{23.6}^{{\rm{o}}}$), respectively. The metasurface is made up of $30\times 30$ different elements. The lengths of $w$ and $l$ of the elements in each row (along the $+x$ direction) are gradually increased and decreased, respectively, but the corresponding dimensions in each column (along the $+y$ direction) are unchanged (Detailed dimensions are list in Supplementary Information). The surface reflected phases distributions of one row along the $x$ direction are shown in Fig. 2(b). Figure 2(c) and (d) show the simulated electro-field distributions of reflected TM and TE waves. And Fig. 2(e) shows the normalized far-field patterns of TM and TE reflected waves. It is clearly demonstrated that the TM and TE reflected waves are deflected to the expected directions of (${\varphi }_{{E}_{x}}\,=\,{0}^{{\rm{o}}}$, ${\theta }_{{E}_{x}}\,=\,{23.6}^{{\rm{o}}}$) and (${\varphi }_{{E}_{y}}\,=\,{180}^{{\rm{o}}}$, ${\theta }_{{E}_{y}}\,=\,-{23.6}^{{\rm{o}}}$).

Then, the type2 metasurface aims to deflect the TM and TE reflected waves to the direction of (${\varphi }_{{E}_{x}}\,=\,{270}^{{\rm{o}}}$, ${\theta }_{{E}_{x}}\,=\,-{23.6}^{{\rm{o}}}$) and (${\varphi }_{{E}_{y}}\,=\,{0}^{{\rm{o}}}$, ${\theta }_{{E}_{y}}\,=\,{23.6}^{{\rm{o}}}$), respectively. We use both of $x$ and $y$ directions to construct metasurface, as shown in Fig. 3(a). Along the $+x$ direction, $w$ is unchanged, and $l$ is gradually increased; while $w$ is gradually decreased, and $l$ is unchanged along the $+y$ direction (Detailed dimensions are list in Supplementary Information). The surface reflected phases distributions for both ${\phi }_{{E}_{x}}(y)$ and ${\phi }_{{E}_{y}}(x)$ along the +x and +y directions are exhibit in Fig. 3(b). Figure 3(c) and (d) show the simulated electro-field distributions. And Fig. 3(e) and (f) show the normalized far-field patterns of TM and TE reflected waves. It is seen that the TM reflected waves are deflected to the (${\varphi }_{{E}_{x}}\,=\,{270}^{{\rm{o}}}$, ${\theta }_{{E}_{x}}\,=\,-{23.6}^{{\rm{o}}}$) direction, and the TE reflected waves are deflected to (${\varphi }_{{E}_{y}}\,=\,{0}^{{\rm{o}}}$, ${\theta }_{{E}_{y}}\,=\,{23.6}^{{\rm{o}}}$) direction. Therefore, according to the simulation results, we conclude that the TM and TE reflected waves can be independently split and deflected, exhibiting good agreements with theoretical results.

**Figure 4:**
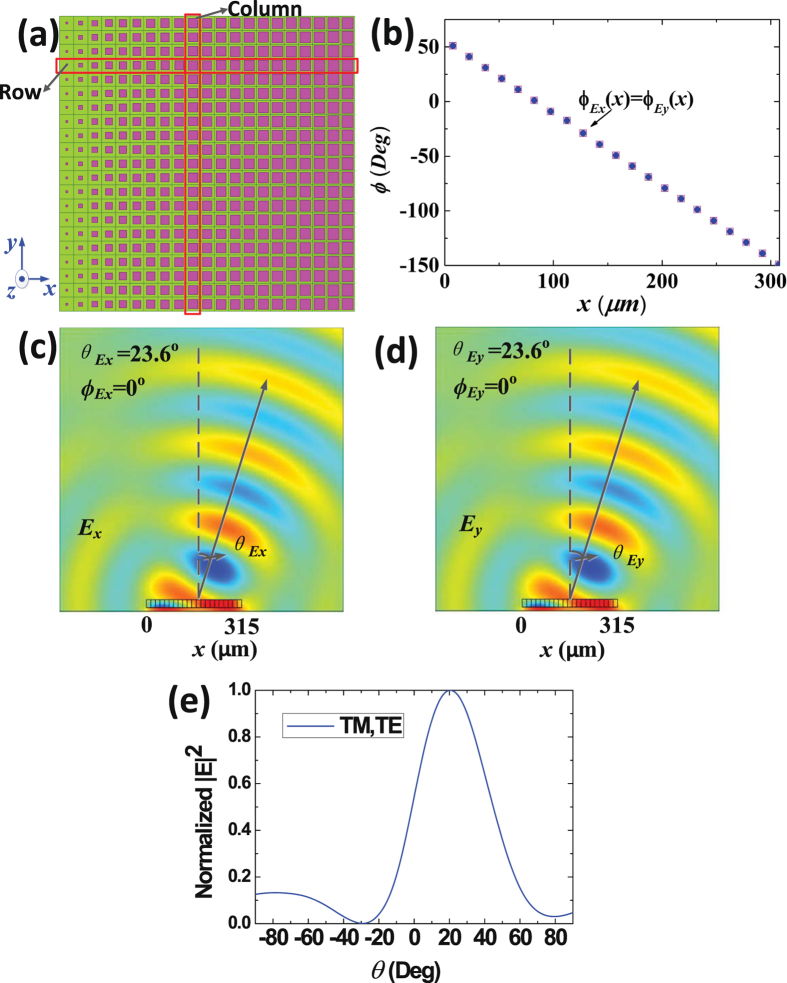
The structure and simulation results of type 3 graphene plasmonic metasurface. (**a**) The top view of the metasurface, which shows that the metasurface is made up of $21\times 21$ elements, and elements in each column are the same. $l$ and $w$ of each element are equal and gradually increased along the $+x$ direction in each row. (**b**) The reflected phases distributions, which demonstrate that ${\phi }_{{E}_{y}}(x)$ and ${\phi }_{{E}_{x}}(x)$ are equally decreased along the $+x$ direction. (**c**) The electric-field distributions of TM reflected waves deflected to the direction of (${\varphi }_{{E}_{x}}\,=\,{0}^{{\rm{o}}}$, ${\theta }_{{E}_{x}}\,=\,{23.6}^{{\rm{o}}}$). (**d**) The electric-field distributions of TE reflected waves deflected to the same direction of (${\varphi }_{{E}_{y}}\,=\,{0}^{{\rm{o}}}$, ${\theta }_{{E}_{y}}\,=\,{23.6}^{{\rm{o}}}$). (**e**) Normalized far-field patterns of TM and TE reflected waves, in the XOZ plane.

Type 3 graphene metasurface is designed to deflect arbitrary reflected linear polarized electromagnetic waves to a specific direction. In principle, the TM and TE components of arbitrary linear polarized waves are reflected to the same direction with same phases. Here, the metasurface is made up of $21\times 21$ elements, and elements in each column are the same, as shown in Fig. 4(a). $l$ and $w$ of each element are equal and gradually increased along the $+x$ direction in each row (Detailed dimensions are list in Supplementary Information). The surface reflection phases distributions along the $+x$ direction are shown in Fig. 4(b). Figure 4(c) and (d) present the simulated electric-field distributions. And Fig. 4(e) shows the normalized far-field patterns of TM and TE reflected waves. It can be seen in the figure that the reflected TM and TE waves are deflected to the same direction of (${\varphi }_{{E}_{x,y}}\,=\,{0}^{{\rm{o}}}$,${\theta }_{{E}_{x,y}}\,=\,{23.6}^{{\rm{o}}}$) with same phases.

**Figure 5:**
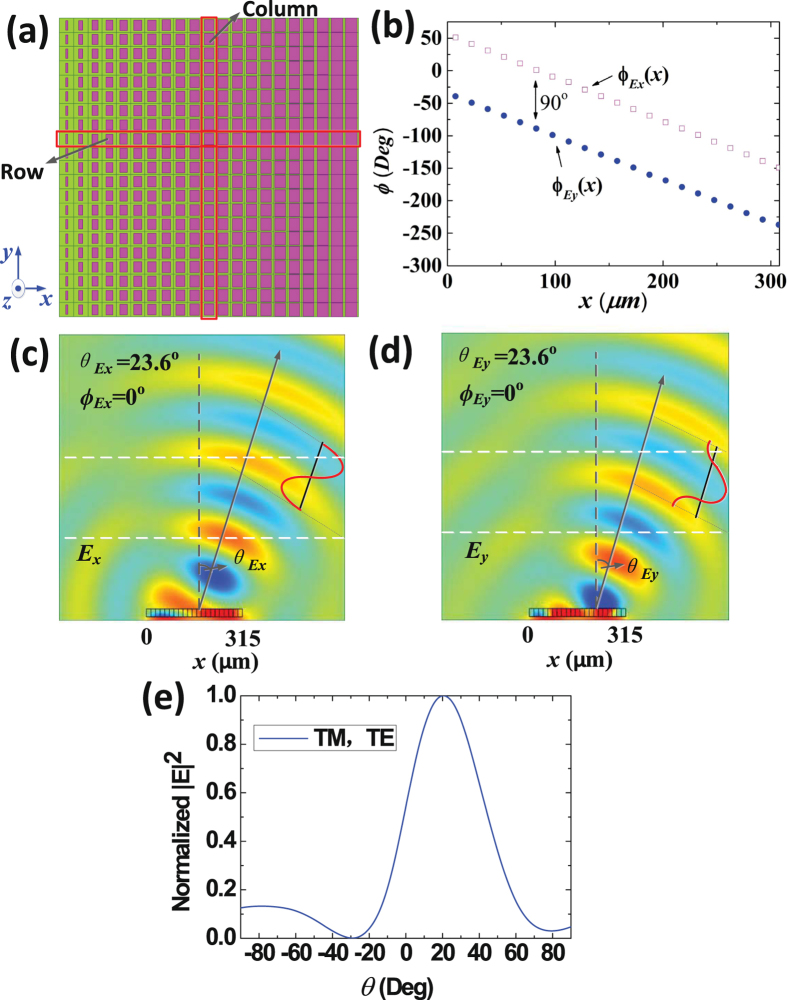
The structure and simulation results of type 4 graphene plasmonic metasurface. (**a**) The top view of the metasurface, which shows that the metasurface is made up of $21\times 21$ elements, and the dimensions $w$ and $l$ of each element are chosen to ensure a ${90}^{{\rm{o}}}$ reflected phase difference, and gradually increased along the $x$ direction. (**b**) The reflected phases distributions, which demonstrate that ${\phi }_{{E}_{y}}(x)$ and ${\phi }_{{E}_{x}}(x)$ have a 90° reflected phase difference and gradually decreased along the $+x$ direction. (**c**) The electric-field distributions of TM reflected waves deflected to the direction of (${\varphi }_{{E}_{x}}\,=\,{0}^{{\rm{o}}}$,${\theta }_{{E}_{x}}\,=\,{23.6}^{{\rm{o}}}$). (**d**) The electric-field distributions of TE reflected waves deflected to the same direction of (${\varphi }_{{E}_{y}}\,=\,{0}^{{\rm{o}}}$,${\theta }_{{E}_{y}}\,=\,{23.6}^{{\rm{o}}}$), whose reflected phase is 90° ahead. (**e**) Normalized far-field patterns of TM and TE reflected waves, in the XOZ plane.

Finally, type 4 is designed to perform as a linear-to-circular polarization converter, which can deflect both TM and TE reflected waves to the same direction with a phase difference of 90°. In this case, the metasurface only contains $x$ direction variation elements(elements in each column are the same). It consists of $21\times 21$ different elements, as shown in Fig. 5(a). The dimensions $w$ and $l$ of each element are chosen to ensure a 90° reflected phase difference, and gradually increased along the $x$ direction (Detailed dimensions are list in Supplementary Information). Figure 5(b) demonstrates the surface reflected phases distributions of ${\phi }_{{E}_{x}}(x)$ and ${\phi }_{{E}_{y}}(x)$ along the $+x$ direction. Figure 5(c) and (d) show the simulated electric-field distributions. And Fig. 5(e) shows the normalized far-field patterns of TM and TE reflected waves. We can see from the figures that the TM and TE reflected waves are deflected to the same directions of (${\varphi }_{{E}_{x,y}}\,=\,{0}^{{\rm{o}}}$, ${\theta }_{{E}_{x,y}}\,=\,{23.6}^{{\rm{o}}}$), and the phase of TM waves is 90° ahead to the TE waves. Therefore, through this proposed graphene metasurface, arbitrary linear-polarized incident waves with normal direction can be reflected to the direction of (${\varphi }_{{E}_{x,y}}\,=\,{0}^{{\rm{o}}}$, ${\theta }_{{E}_{x,y}}\,=\,{23.6}^{{\rm{o}}}$) with the circular polarization.

## Discussion

We have introduced graphene based plasmonic metasurfaces to independently manipulate TM and TE reflected waves in the terahertz regime, overcoming the intrinsic lossy property of metallic metasurface. The graphene patches coupled with a grounded substrate substantially enhance the wave-graphene interaction, achieving almost 300° phase modulation and high reflectivity magnitude. Meanwhile, the reflection phases of both TM and TE waves can be controlled by changing the dimensions of the graphene patches. Finally, based on the general Snell’s law, four types of graphene based metasurfaces with the capability of independently controlling of TM and TE waves have been designed and simulated. The simulation results exhibit excellent performances as theoretical expectations. Therefore, the proposed graphene based plasmonic metasurfaces have good capability to independently control the TM and TE reflected waves with reasonable losses in the terahertz regime.

Meanwhile, considering the practical fabrication, we can design the graphene reflective cell which is consist of 5 layers, such as graphene layer, alumina layer, Polysilicon layer, quartz glasses layer, and ground. The polysilicon can be performed as an electrode. The chemical potential related to the conductivity of graphene can be dynamically tuned by varying the DC voltage (VDC) between the graphene and the polysilicon. Detailed technology to fabricate these kinds of graphene metasurface can be found (in ref. 46), supporting the feasibility of our design.

It is worth emphasizing that the general design procedure formulated herein facilitates further production of such devices for various applications. Considering the existence metasurfaces, the proposed graphene plasmonic metasurface can be applied to realize extremely light-weight, ultra-compact, and high-performances electromagnetic structures for diverse terahertz applications, extending the range of applications even further.

## Methods

### Graphene conductivity modelling

In the terahertz region, the complex surface conductivity $\sigma$ of graphene is dictated by intraband transition. It can be approximated by the Drude model^47^

4 $$\sigma =\frac{{q}_{e}^{2}{k}_{B}T\tau }{\pi {\hslash }^{2}\mathrm{(1}+j\omega \tau )}\times [\frac{{\mu }_{c}}{{k}_{B}T}+2\,\mathrm{ln}({e}^{-\frac{{\mu }_{c}}{{k}_{B}T}}+1)]$$

where ${q}_{e}$ is the elementary charge, ${k}_{B}$ is the Boltzmann’s constant, $\hslash$ is the reduced Plank’s constant. T is temperature, $\tau$ is the relaxation time, $\omega$ is the radian frequency, and ${\mu }_{c}$ is chemical potential. In this paper, the room temperature is set to 300 K, the typical value of relaxation time of graphene is $\tau$ = 1 ps, and the chemical potential ${\mu }_{c}$ is set to 0.2305 eV. Therefore, at the central frequency of 1.2 THz, the calculated impedance of graphene patch is ${Z}_{s}\,=\,\mathrm{1/}\sigma \,=\,37+j279{\rm{\Omega }}$. The surface conductivity can be converted into a volume conductivity ${\sigma }_{v}=\sigma /t$ assuming that $t$ is a very small value^48^. Such an approach has been widely used in many numerical work. However, it imposes significant meshing load and simulation time. Here, we apply transition boundary condition in COMSOL Multiphysics software, which assigns the conductivity to a single interface of a film with finite thickness. As a result, we are able to greatly relieve the meshing difficulty, save memory, and shorten the simulation time.

### Unit-cell modelling

The reflected magnitudes and phases of the proposed element were full-wave simulated using the Floquet’s periodic condition in COMSOL Multiphysics software, taking into account the inter-element coupling. The unit-cell depicted in Fig. 1(b) and (c) was constructed in COMSOL Multiphysics 5.1 software with a top layer graphene patch, deposited on a square grounded quartz glasses (SiO_2_) substrate. The quartz glasses(SiO_2_) substrate has a relative permittivity of ${\varepsilon }_{r}=3.75$, and a loss tangent of tan $\delta =0.0184$. The parameter $w$ and $l$ are the width and length of the graphene patch in the $x$ and $y$ directions, respectively, and $p=15\mu$m denotes unit-cell side-length which also means a periodicity to form the metasurface. And, $t=10nm$ and $d=26\mu$m are the thickness of the metallic ground and quartz glasses (SiO_2_) substrate, respectively. In the simulation, Floquet ports were placed at $z=\pm 3d$ and utilized to characterize the scattering of a normally incident plane wave off the periodic element (the bottom layer of the ground plane was defined as $z=0$ plane) and the reflected magnitudes and phases are obtained by the parametric sweep module of COMSOL 5.1. In detail, the working frequency is set to 1.2 THz, $w$ and $l$ are set as parameters for optimization.

### Graphene plasmonic metasurface simulation

The full-wave simulations of the proposed graphene plasmonic metasurface were performed in COMSOL Multiphysics 5.1 software. According to the specific surface phases distribution, we implement four types of metasurfaces by properly choosing the local element dimensions. To reduce the computation complexity, we apply perfectly matched layer (PML) boundary condition to the rest of the simulation space, ensuring proper numerical evaluation of the fields surrounding the metasurface.

## Electronic supplementary material

Supplementary Information
