# Supplementary material for: Manipulating of Different-Polarized Reflected Waves with Graphene-based Plasmonic Metasurfaces in Terahertz Regime
Source: Sci Rep. 2017 Sep 5;7:10558. doi: 10.1038/s41598-017-10726-y (PMC5585413; doi:10.1038/s41598-017-10726-y)
Supplement: Supplementary file 1 — Supplementary Information [file 41598_2017_10726_MOESM1_ESM.doc]

**Supplementary Information**

**Title:** **Manipulating of Different-Polarized Reflected Waves with Graphene-based Plasmonic Metasurfaces in Terahertz Regime**

Li Deng^1,*^, Yongle Wu^2^, Chen Zhang^1^, Weijun Hong^1^, Biao Peng^1^ , Jianfeng Zhu^1^ and Shufang Li^1^

^1^ Beijing Key Laboratory of Network System Architecture and Convergence, School of Information and Communication Engineering. Beijing University of Posts and Telecommunications, P. O. Box. 282, 100876, Beijing, China.

^2^ Beijing Key Laboratory of Work Safety Intelligent Monitoring, School of Electronic Engineering, Beijing University of Posts and Telecommunications, P. O. Box. 282, 100876, Beijing, China.

* Corresponding Author. E-mail: dengl@bupt.edu.cn

1. **Graphene Metasurfaces for Polarized Beam Splitting ( Type 1 )**

In the simulation we set 30$\times$30 graphene patches with periodicity of 15 μm in both *x* and *y* directions. The phase shift between two adjacent unit cells in *x* direction is nearly 10^o^. Then from Figure. 1(d), we choose suitable patches that can provide these phase differences in the *x* direction, then copy these 30 patches 30 times along the +*y* direction, and form a 30$\times$30 elements metasurface. All data are shown in the following table.

| No. in the +*x* direction | Δphase of E_x_ (Deg.) | patch width *w*(μm) | Δphase of E_y_  (Deg.) | patch length *l*(μm) |
| --- | --- | --- | --- | --- |
| 1 | 51.02868 | 2 | -237.173 | 14.99 |
| 2 | 41.02043 | 4.18 | -229.157 | 14.87 |
| 3 | 31.03598 | 6 | -219.181 | 14.54 |
| 4 | 21.01676 | 7.12 | -208.96 | 14.16 |
| 5 | 11.02839 | 7.91 | -199.039 | 13.74 |
| 6 | 1.0131 | 8.51 | -189.047 | 13.38 |
| 7 | -9.12464 | 8.95 | -179.109 | 13.06 |
| 8 | -17.3936 | 9.23 | -168.928 | 12.76 |
| 9 | -29.0925 | 9.52 | -159.999 | 12.53 |
| 10 | -39.2902 | 9.82 | -149.113 | 12.26 |
| 11 | -49.171 | 10.07 | -139.005 | 12.02 |
| 12 | -58.9787 | 10.31 | -128.876 | 11.81 |
| 13 | -69.1597 | 10.57 | -119.059 | 11.6 |
| 14 | -79.2615 | 10.79 | -109.056 | 11.4 |
| 15 | -88.9184 | 10.98 | -98.8588 | 11.18 |
| 16 | -98.8588 | 11.18 | -88.9184 | 10.98 |
| 17 | -109.056 | 11.4 | -79.2615 | 10.79 |
| 18 | -119.059 | 11.6 | -69.1597 | 10.57 |
| 19 | -128.876 | 11.81 | -58.9787 | 10.31 |
| 20 | -139.005 | 12.02 | -49.171 | 10.07 |
| 21 | -149.113 | 12.26 | -39.2902 | 9.82 |
| 22 | -159.999 | 12.53 | -29.0925 | 9.52 |
| 23 | -168.928 | 12.76 | -17.3936 | 9.23 |
| 24 | -179.109 | 13.06 | -9.12464 | 8.95 |
| 25 | -189.047 | 13.38 | 1.0131 | 8.51 |
| 26 | -199.039 | 13.74 | 11.02839 | 7.91 |
| 27 | -208.96 | 14.16 | 21.01676 | 7.12 |
| 28 | -219.181 | 14.54 | 31.03598 | 6 |
| 29 | -229.157 | 14.87 | 41.02043 | 4.18 |
| 30 | -237.173 | 14.99 | 51.02868 | 2 |

1. **Graphene Metasurfaces for Polarized Beam Splitting ( Type 2)**

In the simulation we set 30$\times$30 graphene patches with periodicity of 15 μm in both x and y directions. The phase shift between two adjacent unit cells is nearly 10^o^. Then from Figure. 1(d), we choose suitable patches that can provide these phase differences, and form a 30$\times$30 lelments metasurface. It is worth noting that the width *w* is unchanged and length *l* is gradually increased along the +*x* direction, meanwhile, *w* is gradually decreased and *l* is unchanged along the +*y* direction. All data are shown in the following table.

| No. in the +*x* direction | Δphase of E_y_ (Deg.) | patch length *l*(μm) | No. in the +*y* direction | Δphase of E_x_  (Deg.) | patch width *w*(μm) |
| --- | --- | --- | --- | --- | --- |
| 1 | 51.02868 | 2 | 1 | -237.173 | 14.99 |
| 2 | 41.02043 | 4.18 | 2 | -229.157 | 14.87 |
| 3 | 31.03598 | 6 | 3 | -219.181 | 14.54 |
| 4 | 21.01676 | 7.12 | 4 | -208.96 | 14.16 |
| 5 | 11.02839 | 7.91 | 5 | -199.039 | 13.74 |
| 6 | 1.0131 | 8.51 | 6 | -189.047 | 13.38 |
| 7 | -9.12464 | 8.95 | 7 | -179.109 | 13.06 |
| 8 | -17.3936 | 9.23 | 8 | -168.928 | 12.76 |
| 9 | -29.0925 | 9.52 | 9 | -159.999 | 12.53 |
| 10 | -39.2902 | 9.82 | 10 | -149.113 | 12.26 |
| 11 | -49.171 | 10.07 | 11 | -139.005 | 12.02 |
| 12 | -58.9787 | 10.31 | 12 | -128.876 | 11.81 |
| 13 | -69.1597 | 10.57 | 13 | -119.059 | 11.6 |
| 14 | -79.2615 | 10.79 | 14 | -109.056 | 11.4 |
| 15 | -88.9184 | 10.98 | 15 | -98.8588 | 11.18 |
| 16 | -98.8588 | 11.18 | 16 | -88.9184 | 10.98 |
| 17 | -109.056 | 11.4 | 17 | -79.2615 | 10.79 |
| 18 | -119.059 | 11.6 | 18 | -69.1597 | 10.57 |
| 19 | -128.876 | 11.81 | 19 | -58.9787 | 10.31 |
| 20 | -139.005 | 12.02 | 20 | -49.171 | 10.07 |
| 21 | -149.113 | 12.26 | 21 | -39.2902 | 9.82 |
| 22 | -159.999 | 12.53 | 22 | -29.0925 | 9.52 |
| 23 | -168.928 | 12.76 | 23 | -17.3936 | 9.23 |
| 24 | -179.109 | 13.06 | 24 | -9.12464 | 8.95 |
| 25 | -189.047 | 13.38 | 25 | 1.0131 | 8.51 |
| 26 | -199.039 | 13.74 | 26 | 11.02839 | 7.91 |
| 27 | -208.96 | 14.16 | 27 | 21.01676 | 7.12 |
| 28 | -219.181 | 14.54 | 28 | 31.03598 | 6 |
| 29 | -229.157 | 14.87 | 29 | 41.02043 | 4.18 |
| 30 | -237.173 | 14.99 | 30 | 51.02868 | 2 |

1. **Graphene Metasurfaces for Linear Polarized Wave Deflection (Type 3)**

In the simulation we set 21$\times$21 graphene patches with periodicity of 15 μm in both x and y directions. The phase shift between two adjacent unit cells is nearly 10^o^. Then from Figure. 1(d), we choose suitable patches that can provide these phase differences, and form a 21$\times$21 elements metasurface. It is worth noting that the elements in each column are the same, so *l* and *w* are equal and gradually increased along the +*x* direction in each row. All data are shown in the following table.

| No. in the +*x* direction | Δphase of E_x_ (Deg.) | patch width *w*(μm) | Δphase of E_y_  (Deg.) | patch length *l*(μm) |
| --- | --- | --- | --- | --- |
| 1 | 51.02868 | 2 | 51.02868 | 2 |
| 2 | 41.02043 | 4.18 | 41.02043 | 4.18 |
| 3 | 31.03598 | 6 | 31.03598 | 6 |
| 4 | 21.01676 | 7.12 | 21.01676 | 7.12 |
| 5 | 11.02839 | 7.91 | 11.02839 | 7.91 |
| 6 | 1.0131 | 8.51 | 1.0131 | 8.51 |
| 7 | -9.12464 | 8.95 | -9.12464 | 8.95 |
| 8 | -17.3936 | 9.23 | -17.3936 | 9.23 |
| 9 | -29.0925 | 9.52 | -29.0925 | 9.52 |
| 10 | -39.2902 | 9.82 | -39.2902 | 9.82 |
| 11 | -49.171 | 10.07 | -49.171 | 10.07 |
| 12 | -58.9787 | 10.31 | -58.9787 | 10.31 |
| 13 | -69.1597 | 10.57 | -69.1597 | 10.57 |
| 14 | -79.2615 | 10.79 | -79.2615 | 10.79 |
| 15 | -88.9184 | 10.98 | -88.9184 | 10.98 |
| 16 | -98.8588 | 11.18 | -98.8588 | 11.18 |
| 17 | -109.056 | 11.4 | -109.056 | 11.4 |
| 18 | -119.059 | 11.6 | -119.059 | 11.6 |
| 19 | -128.876 | 11.81 | -128.876 | 11.81 |
| 20 | -139.005 | 12.02 | -139.005 | 12.02 |
| 21 | -149.113 | 12.26 | -149.113 | 12.26 |

1. **Graphene Metasurfaces for Linear to Circular Polarization Convertor (Type 4)**

In the simulation we set 21$\times$21 graphene patches with periodicity of 15 μm in both *x* and *y* directions. The phase shift between two adjacent unit cells is nearly 10^o^, meanwhile, the dimensions *w* and *l* of each element are chosen to ensure a 90^o^ reflected phase difference. Then from Figure. 1(d), we choose suitable patches that can provide these phase differences, and form a 21$\times$21 elements metasurface. All data are shown in the following table.

| No. in the +*x* direction | Δphase of E_x_ (Deg.) | patch width *w*(μm) | Δphase of E_y_  (Deg.) | patch length *l*(μm) |
| --- | --- | --- | --- | --- |
| 1 | 51.02868 | 2 | -39.2902 | 9.82 |
| 2 | 41.02043 | 4.18 | -49.171 | 10.07 |
| 3 | 31.03598 | 6 | -58.9787 | 10.31 |
| 4 | 21.01676 | 7.12 | -69.1597 | 10.57 |
| 5 | 11.02839 | 7.91 | -79.2615 | 10.79 |
| 6 | 1.0131 | 8.51 | -88.9184 | 10.98 |
| 7 | -9.12464 | 8.95 | -98.8588 | 11.18 |
| 8 | -17.3936 | 9.23 | -109.056 | 11.4 |
| 9 | -29.0925 | 9.52 | -119.059 | 11.6 |
| 10 | -39.2902 | 9.82 | -128.876 | 11.81 |
| 11 | -49.171 | 10.07 | -139.005 | 12.02 |
| 12 | -58.9787 | 10.31 | -149.113 | 12.26 |
| 13 | -69.1597 | 10.57 | -159.999 | 12.53 |
| 14 | -79.2615 | 10.79 | -168.928 | 12.76 |
| 15 | -88.9184 | 10.98 | -179.109 | 13.06 |
| 16 | -98.8588 | 11.18 | -189.047 | 13.38 |
| 17 | -109.056 | 11.4 | -199.039 | 13.74 |
| 18 | -119.059 | 11.6 | -208.96 | 14.16 |
| 19 | -128.876 | 11.81 | -219.181 | 14.54 |
| 20 | -139.005 | 12.02 | -229.157 | 14.87 |
| 21 | -149.113 | 12.26 | -237.173 | 14.99 |
